# Comb-like PEG-containing polymeric composition as low toxic drug nanocarrier

**DOI:** 10.1186/s12645-018-0045-5

**Published:** 2018-12-20

**Authors:** Lesya Kobylinska, Igor Patereha, Natalia Finiuk, Natalia Mitina, Anna Riabtseva, Igor Kotsyumbas, Rostyslav Stoika, Alexander Zaichenko, Sandor G. Vari

**Affiliations:** 1 Danylo Halytsky Lviv National Medical University, Pekarska str., 69a, Lviv, 79010 Ukraine; 2 State Scientific-Research Control Institute of Veterinary Medicinal Products and Feed Additives, Donetska str., 11, Lviv, 79019 Ukraine; 3 Institute of Cell Biology, Drahomanov str., 14/16, Lviv, 79005 Ukraine; 4 Lviv Polytechnic National University, S. Bandera str., 12, Lviv, 79013 Ukraine; 5 International Research and Innovation in Medicine Program, Cedars-Sinai Medical Center, 6500 Wilshire Blvd., Ste. 2102, Los Angeles, CA 90048-5502 USA

**Keywords:** Drug delivery system, Polyethylene glycol, Polymeric nanocarrier, Toxicity, Rats, Mice

## Abstract

**Background:**

Development of biocompatible multifunctional polymeric drug carriers is crucial in modern pharmaceutics aimed to create “smart” drugs. The high potential of the PEGylated comb-like polymeric nanocarrier (PNC) in delivering both traditional and experimental drugs to tumor cells in vitro and in vivo has been demonstrated previously. In the present study, we investigated the general toxicity of polyethylene glycol (PEG) processed with both covalent and non-covalent attachments of PEG to compose a comb-like polymer that behaves like a simple chain of n monomers decorated with swollen side chains. The PNC possesses properties of a water-soluble surfactant containing methyl-terminated PEG side branches in some monomer units attached covalently to the carbon chain backbone.

**Results:**

We have demonstrated that the synthesized PNC possesses weak toxic effects toward human leukemia cells (HL-60 and Jurkat lines), as well as toward hepatocellular (HepG2), colon (HCT116) and breast (MCF-7) tumor cell lines. Additionally, after a long period (20 days) of intraperitoneal administration, the PNC had no significant toxic effects in laboratory white mice (470 mg/kg body mass in 1 ml) and Wistar rats (440 mg/kg body mass in 10 ml).

**Conclusion:**

The developed PNC we studied can be qualified as a compound of grade 4 toxicity (low toxicity substance). The reduced toxicity of this PNC in combination with its improved bioavailability and previously detected capability to enhance cytotoxicity toward tumor cells in vitro and potential tumor treatment effects in vivo suggests its potential as a safe drug delivery platform for treating various diseases, especially cancer.

## Introduction

In the last two decades, lipid and polymer-based nanocarriers have been studied as alternative drug delivery systems that can enhance the solubility of drugs by encapsulating existing and new anticancer drugs (Feng et al. 2015; Zhang et al. 2009). Nanocarriers provide additional advantages for tumor treatment since the conjugate of the anticancer drug with different carriers may reduce systemic toxicity and enhance drug accumulation in malignant tissue (Feng et al. 2015; Zhang et al. 2009). In these ways, nanocarrier-based drug delivery will improve the efficacy of anticancer chemotherapy (Wang et al. 2017).

In modern pharmacology, polymeric nanocarriers (PNCs) are the most promising drug delivery platforms for anticancer drugs (Han et al. 2018). PNCs have specific physicochemical properties that should be considered during drug delivery (e.g., molecular weight and size, solubility, density, specific gravity, pH, dissociation) to enhance biocompatibility and biodegradability, and with additional functionalization make drug delivery more effective (Han et al. 2018; Heffeter et al. 2013; Riabtseva et al. 2012; Nath Roy et al. 2017; Li et al. 2003; Zhang et al. 2009).

In the evolution of modern biopharmaceuticals, some of the main tasks are to develop safe nanosized carriers that have low toxicity, physical stability in blood, compatibility with metabolites of the organism, also controlled impact on cells’ damage, and the potential to improve targeted delivery of anticancer drugs to tumors (Nath Roy et al. 2017; Igarashi 2008; Zhang et al. 2009; Senkiv et al. 2014). Polyethylene glycol (PEG) is a non-ionic compound that is often used for this purpose (Heffeter et al. 2013; Riabtseva et al. 2012; Nath Roy et al. 2017). Studies have shown that the conjugation of antitumor drugs with a PNC reduced the toxicity and improved the pharmacokinetic parameters of the drugs and increased their therapeutic efficacy (Heffeter et al. 2013; Igarashi 2008). Polymer-based therapeutics has been established as an innovative and dependable method, since the PNC can conjugate with a wide variety of molecules and compounds (e.g., proteins, enzymes, nanoparticles, liposomes, low molecular weight drugs) (Wang et al. 2017).

PEG has been studied comprehensively as a drug carrier because it is soluble in both organic and hydrophilic solvents. Unlike many other synthetic polymers, PEG is relatively hydrophilic. Conjugation with PEG increases the solubility of hydrophobic drugs and prolongs the circulation time in the organism. PEG also minimizes the nonspecific absorption of the drug, provides specific affinity toward the targeted tumor, and increases the drug accumulation in malignant tissue (Heffeter et al. 2013; Riabtseva et al. 2012; Nath Roy et al. 2017). PEG can be conjugated to other polymers to make them less hydrophobic (i.e., PEGylation). The changes in surface hydrophilicity prevent protein adsorption, thereby enabling cell adhesion and proliferation on biomaterial scaffolds (Bunker 2012; Feng et al. 2015). PEG availability in a PNC is a valuable characteristic that makes possible the formation of micelles to make an aggregate of molecules in a colloidal solution, thus containing the hydrophobic compound (e.g., 4-thiazolidinone derivative) and creating a hydrophilic environment for drug delivery in the living organism (Bunker 2012; Feng et al. 2015; Heffeter et al. 2013).

However, the toxicological aspects of using polymeric carriers in pharmacy and medicine remain poorly understood (Faqi 2013, 2017; Bobo et al. 2016). Changes in the attached PEG molecules will modify the shape and size of nanoparticles and alter the toxicity of the PNCs. Such changes will influence the metabolism in research animals (Pinto Reis et al. 2006). The biosafety of novel drugs immobilized on various carriers is uncertain and limits their use in medical practice (Bobo et al. 2016). Therefore, defining the biosafety of different drug delivery systems, particularly their toxicity, is an important stage in the development of new therapeutics.

Previously, high efficiency of using PNCs has been demonstrated for delivery of doxorubicin (Senkiv et al. 2014), ruthenium-containing antitumor preparation KP-1019 (Heffeter et al. 2013), and experimental anticancer 4-thiazolidinone derivatives (Kobylinska et al. 2015, 2016) to inhibit growth and survival of tumor cells in vitro and in tumor-bearing laboratory mice. The main goal of the present study was to estimate the indicators of general toxicity of the applied PNC in laboratory animals (rats and mice).

## Materials and methods

**Fig. 1:**
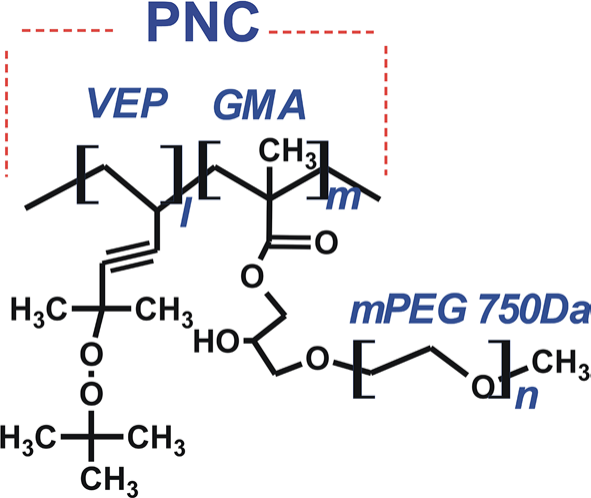
General structure of the polymeric nanocarrier (PNC): “l”, “m”, and “n” indicate the number of structural components of the PNC listed in Table 1

As shown in Fig. 1, PNC is a water-soluble comb-like polymer of poly(VEP-co-GMA)-*graft*-mPEG consisting of a backbone copolymer of 5-(*tert*-butylperoxy)-5-methylhex-1-en-3-yne (VEP, denoted by “l”) and glycidyl methacrylate (GMA, denoted by “m”), and grafted side PEG chains (PEG denoted by “n”) (Riabtseva et al. 2012).

### Synthesis and characteristics of PNC

The synthesis of the PNC was carried out as described previously (Heffeter et al. 2013; Riabtseva et al. 2012; Senkiv et al. 2014). The composition of the PNC was calculated from the results of elemental analysis and analysis of functional groups (Steyermark 1961; Voronov et al. 1996).

The PNC water solution was prepared as follows: 0.093 g of PNC was dissolved in 0.9 ml DMSO and this solution was added to 8.5 ml of saline solution (0.9% aqueous NaCl solution). Then, the solution was stirred for 1 h and sonicated for 20 s, [PNC] = 9.9 mg/ml.

Transmission electron microscopy (TEM) and scanning electron microscopy (SEM) studies of the morphology of the micelles: a transmission electron microscope JEM-200A (JEOL, Japan) was used at an accelerating voltage of 200 kV. The samples were prepared via polymer dissolution in water, as described above. Samples were prepared by spraying tested solution on a substrate using the ultrasonic dispersant UZDN-1A (Ukrrospribor Ltd, Ukraine) that produces a uniform coating on the substrates. A thin amorphous carbon film deposited on a copper grid was used as a substrate. The dispersant options used were a type UZDN-1 possessing power of 50 W and frequency of 35 kHz. A Zeiss Supra 40/40VP scanning electron microscope (Carl Zeiss Group, Germany) was also used in this study.

The size of polymer micelles was measured by dynamic light scattering (DLS) using a Zetasizer Nano ZS instrument (Malvern Instruments GmbH, Stuttgart, Germany) and by photon correlation spectra, using the NIBS (Non-Invasive Back Scatter) technology at 25 °C. The samples for DLS measurements were prepared by dissolution of polymers in bidistilled water, pH 6.5–7.0, and the copolymer concentration was 1∙10^−2^–5∙10^−2^ g/ml. Three to five measurements were made for every sample (each measurement consisted of five cycles, the range between measurements was 5 min). For temperature-dependent DLS studies, the solution was allowed to equilibrate for at least 1 h prior to data collection. Zeta potential experiments were carried out with a Zetasizer Nano Particle (Malvern Instruments GmbH, Stuttgart, Germany) at a fixed temperature of 25 °C. The distributions of the hydrodynamic diameter of PNC at different temperatures were conducted on the Nano Particle Analyzer SZ-100 (HORIBA, Ltd., Kyoto, Japan) and the measurements show a dependence of hydrodynamic diameter of PNC on temperature.

### Cytotoxicity in in vitro MTT assay

Human myeloid leukemia HL-60, human hepatocarcinoma HepG2 cells, and human colon carcinoma HCT116 cells were used from the Institute of Cancer Research at Vienna Medical University (Vienna, Austria). Human breast adenocarcinoma MCF-7 and human T cell leukemia Jurkat cells were obtained from the Institute of Experimental Pathology, Oncology and Radiobiology (Kyiv, Ukraine). Cells were grown in RPMI-1640 culture medium (APP, Austria) or Dulbecco’s modified Eagle’s medium (DMEM, Sigma-Aldrich, USA) supplemented with 10% fetal bovine serum (Biowest, France) at the standard conditions.

The cytotoxicity was measured using colorimetric MTT assay (EZ4U, Biomedica, Austria) for assessing cell functional activity after treatment with the PNC. Cells were plated at 5000 cells/well (substrate-dependent cells) or 15,000 cells/well (suspension cells) in 100 µl in 96-well plates and allowed to incubate overnight. PNC (equivalent to the amount of polymer in complex with the anticancer drug) was added in 100 µl of culture medium and the cells were incubated for 72 h. Afterward, MTT assay was performed according to the manufacturer’s recommendations (EZ4U, Biomedica, Austria). Briefly, 20 μl of dye solution was added to 200 μl of cell culture and incubated for the next 1–4 h at 37 °C. The optical density was measured with the Absorbance Reader BioTek ELx800 (BioTek Instruments, Inc., USA) at 490 nm with 630 nm as a reference.

#### Trypan blue exclusion test

Cells were plated at 50,000 cells/well (substrate-dependent cells) or 500,000 cells/well (suspension cells) in 1 ml in 24-well plates and allowed to incubate overnight. PNC at different concentrations was added to the cell culture and incubated for the next 72 h. The cell number was counted in a hemocytometric Neubauer chamber after staining with Trypan blue dye (DV-T10282, Invitrogen, Life Technologies Corporation) at 0.04% final concentration. The percentage of live cells related to control was calculated as the cell number experiment/cell number control × 100%.

#### Data analyses

The results of MTT and Trypan blue assays are presented as the mean (M) ± standard deviation (SD) of three replications. The data were analyzed and illustrated using GraphPad Prism 6 software (GraphPad Software, La Jolla, CA, USA).

### Evaluation of PNC toxicity in vivo

All animal studies were conducted according to the European Convention on Protection of Vertebrate Animals (Strasbourg, 1986) and corresponding Law of Ukraine (N944, 14.12.2009). The protocols of this study and experimental procedures were approved by the Ethical Committee at Lviv National Medical University (N2, 16.02.2015). The experiments were carried out on white laboratory mice 3–4 months old with body weight of 18–24 g and white Wistar rats 3–4 months old with body weight of 143–255 g. The PNC was injected daily into the peritoneal cavity. In total, 36 rats and 36 mice were used in the study. Mice were injected with the PNC at doses of 0.1, 0.3, 0.5, and 1.0 ml; in rats, the doses were 1.0, 3.0, 5.0, and 10.0 ml. The highest dose of the PNC was repeatedly administered to six rats (10.0 ml) and six mice (1.0 ml). 1 ml of the solution contained 9.9 mg of the PNC. We could not further increase the amount of applied substance because of the critical volume used for injection. Control groups of rats and mice were injected with a physiological solution of the same volume. To avoid application of too large volume of the diluted substance (20 mL/kg of body weight of laboratory rats), we injected the liquid in two steps during 30 min (Diehl et al. 2001).

Weighing of experimental animals was carried out after 5, 8, and 14 days. After administration of the PNC, laboratory animals were monitored for 14 days. The following indicators were considered: appearance, uniqueness of animal behavior, intensity and character of motor activity, assessment of food and water consumption, mass of animals, condition of fur and visible mucous membranes, respiration rate, time of occurrence and nature and severity of intoxication, as well as the time of death of the animals or their recovery (Faqi 2013, 2017).

The animals (rats) were euthanized by decapitation while under thiopental anesthesia on the 20th day. Blood was collected and used to obtain serum for determination of the activities of alkaline phosphatase (ALP; 3.1.3.1), α-amylase (3.2.1.1), γ-glutamyltransferase (GGT; 2.3.2.2), lactate dehydrogenase (LDH; 1.1.1.27), alanine transaminase (ALT; 2.6.1.2), aspartate transaminase (AST; 2.6.1.1), and creatine phosphokinase (2.7.3.2). The concentrations of total protein, glucose, urea, creatinine, and of calcium, iron, sodium and chloride ions were also measured. These parameters were measured with standard kits for an automated biochemistry analyzer (Humalyzer 3000, Germany).

Daily urine and feces were collected from treated rats, dried, and examined using thin-layer chromatography on a Sorbfil plate (Russian Federation) in acetone–dioxan (4:1) solvent mixture. The chromatographic plates were developed with iodine vapor, and pure VEP, GMA, and PEG were used as controls for the presence of specific compounds.

The results were established by colorimetric assay using the Absorbance Reader (BioTek Instruments, Inc., Winooski, VT, USA). Data analysis was performed with GraphPad Prism 6 software (GraphPad Software). Statistical analysis of the in vivo results was done using variance (MS Excel software; Microsoft Corp., Redmond, WA, USA) and Student’s *t* test. The difference was considered statistically significant and marked with stars for: * *P* ≤ 0.05.

## Results

### Some colloidal chemical properties of the PNC

The structure of the synthesized polymers was confirmed by the analysis of functional groups (Table 1), as well as NMR studies. The absence of the residual epoxy groups in the resulting product was confirmed by ^1^H-NMR spectroscopy (Bruker, Billerica, MA, US). ^1^H-NMR spectra were recorded on a Bruker Avance DPX 300 spectrometer at 300.13 MHz. The structure of the PNC in the DMSO-d^3^ was confirmed by ^1^H NMR. In the ^1^H-NMR spectroscopy, the polymeric skeletal CH_2_ and CH were at 1.66 ppm and 3.64 ppm, respectively; the GMA–*graf–*PEG fragment units demonstrated the following signals: CH_3_–C–C(O)–O– at 1.12 ppm, CH_3_–O–CH_2_–CH_2_– at 3.24 ppm, –CH_2_–CH_2_-O– at 3.65 ppm; –O-CH_2_–CH(OH)– at 4.67 ppm. The VEP fragment units demonstrated the following signals: (CH_3_)_3_–C–O–O– at 1.18 ppm and –O–O(CH_3_)_2_– at 1.36 ppm.

**Table 1:** Composition, molecular weight, and colloidal chemical characteristics of the PNC

Composition of PNC, %			PNC molar weight, kDa	Characteristics of micelle-like structures			
				DLS (± SD) Z-average hydrodynamic diameter, nm	TEM (± SD) Average diameter, nm	SEM Average diameter, nm	Zeta potential, mV
VEP (*l*)	GMA (*m*)	PEG (*n*)					
1.4	69.1	29.5	245.0	50.2 (± 25.0)	35.0 (± 6.5)	45.0 (± 15.2)	0.11

Table 1 shows the composition of the PNC and the size of the micelle-like colloidal structures, which were determined by various methods.

**Fig. 2:**
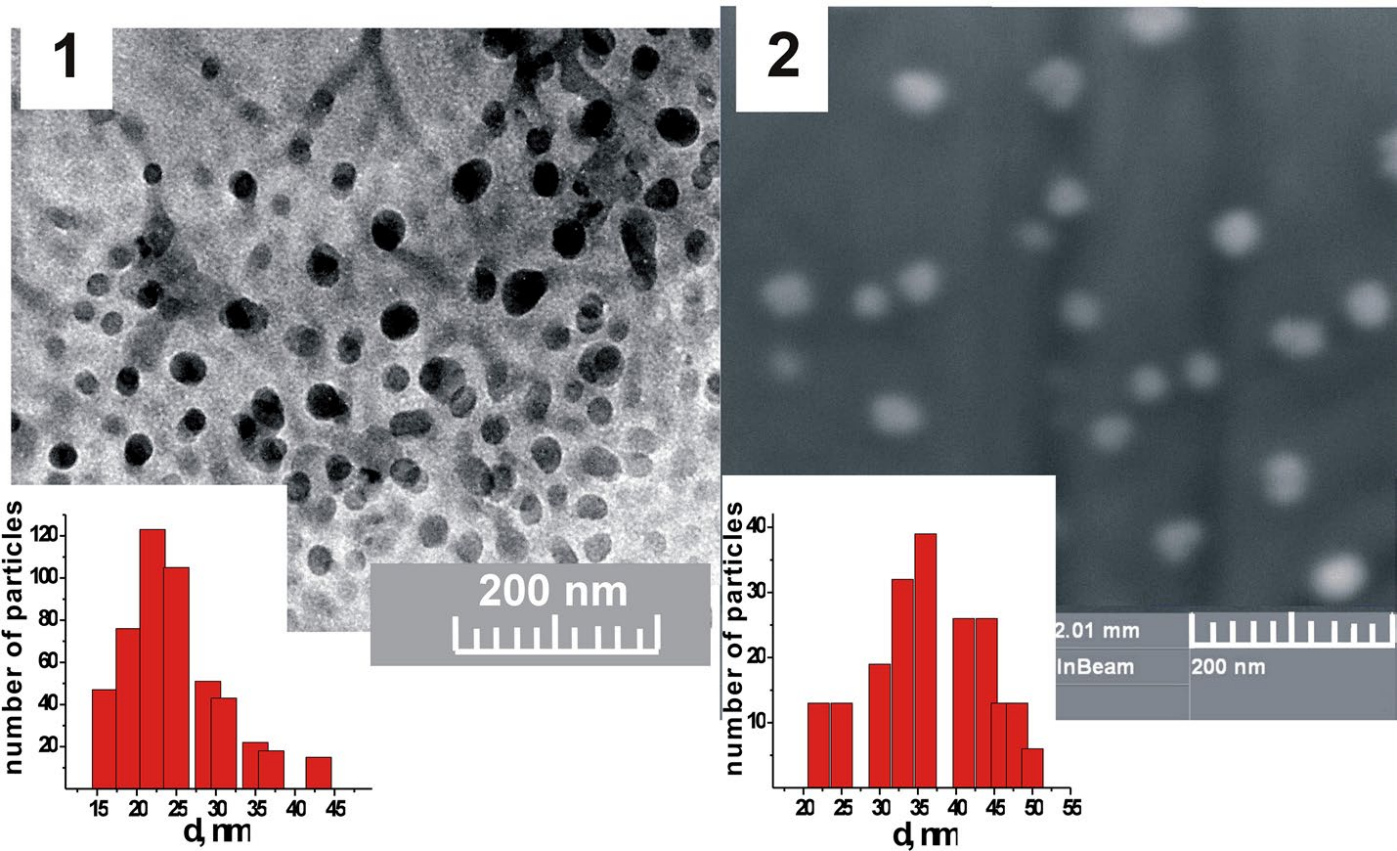
TEM (1) and SEM (2) images of the polymeric nanocarrier (PNC)

**Fig. 3:**
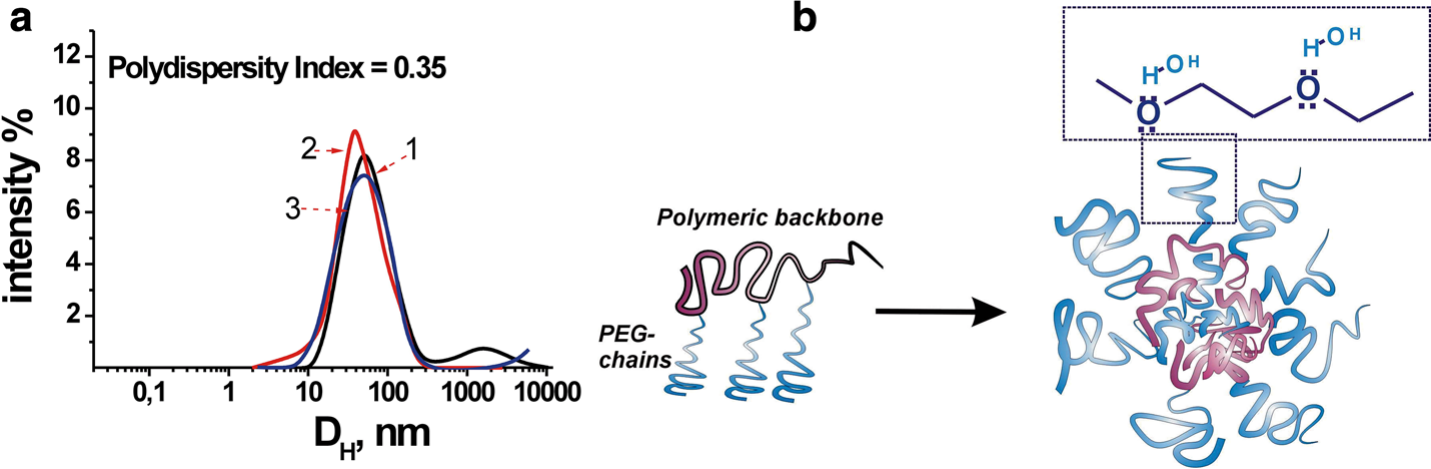
Hydrodynamic diameter distributions of PNC formed by poly(VEP-co-GMA)-*graft*-mPEG(750) (measurements made at intervals of 5 min: 1—at once, 2—in 5 min, 3—in 10 min) (**a**) and schematic structure of the micelles (**b**)

The diameter of the PNC-formed micelle-like structures was 30–45 nm according TEM and SEM data (Fig. 2). It is obvious from the intensity results of the DLS measurements (Fig. 3) that the values of the PNC size are in the range 20–200 nm with an average hydrodynamic diameter of ~ 50 nm. As expected, the hydrodynamic diameters from the DLS measurements were substantially larger compared to the dimensions determined by the TEM and SEM methods.

**Fig. 4:**
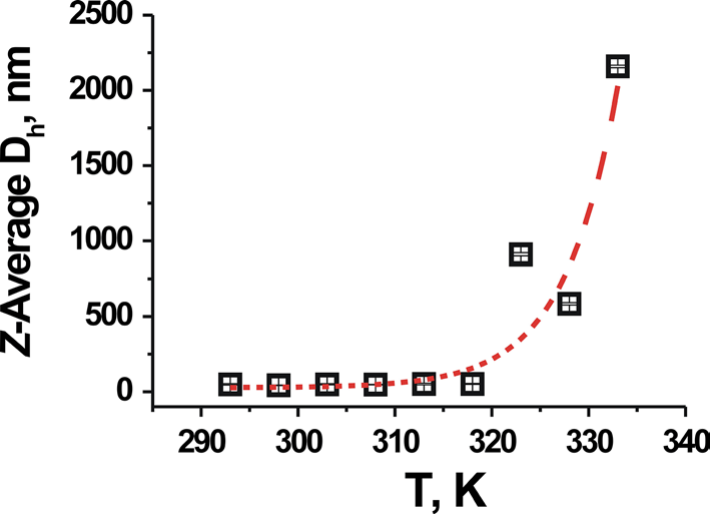
The dependence of the hydrodynamic diameter of PNC formed by the poly(VEP-co-GMA)-*graft*-mPEG(750) on temperature

As seen in Fig. 4, it is apparent that the PNC size does not change in the temperature range of 293–318 К, and that the dispersion is highly stable until 320 К. Large agglomerates are formed at 333 К and sedimentation of the polymer aggregates is observed.

### Evaluation of PNC cytotoxicity in vitro

**Fig. 5:**
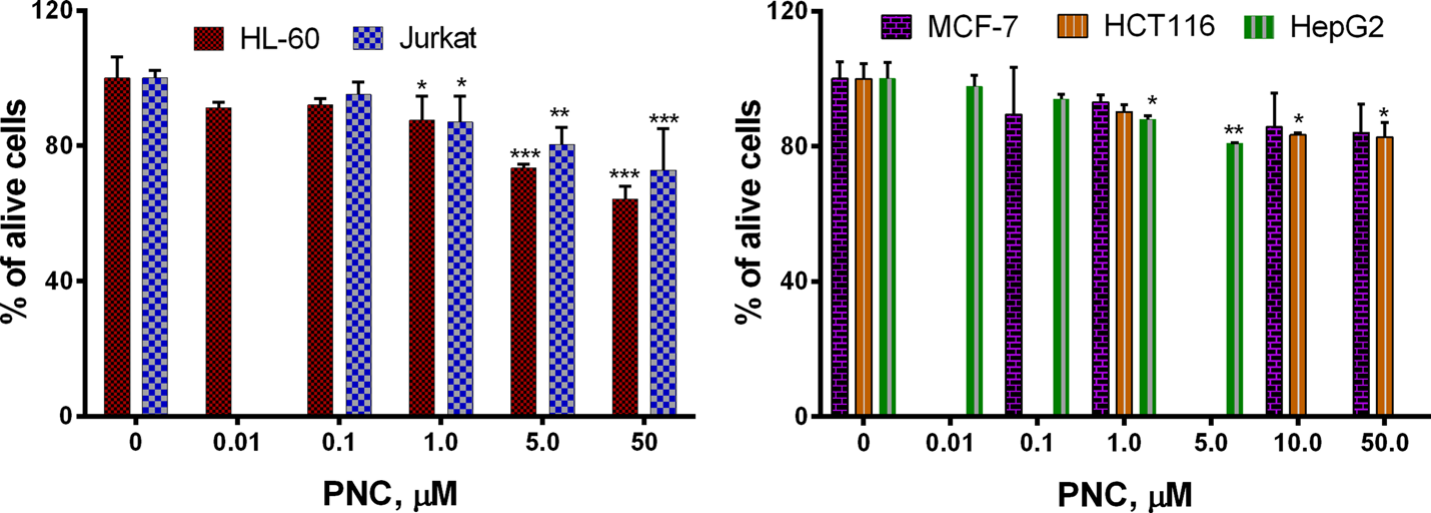
Values of IC_50_ determined by MTT assay after treatment for 72 h of these cell lines with the PNC: human leukemia (HL-60, Jurkat), hepatocellular (HepG2), colon (HCT116), and breast (MCF-7) tumor. *P ≤ 0.05; **P ≤ 0.01 (difference compared with the non-treated control cells)

PNC showed a weak toxic effect in MTT assay toward human leukemia (HL-60, Jurkat), hepatocellular (HepG2), colon (HCT116), and breast (MCF-7) tumor cell lines. PNC at the highest dose of 50 µM inhibited HL-60 cell viability by 35.7% and the growth of Jurkat cells by 27.2%. PNC at 5 µM reduced cell growth of HepG2 cells by 19%. At the 50 µM dose of PNC, the growth inhibition of MCF-7 cells was 16% and HCT116 cells was 17% (Fig. 5).

**Fig. 6:**
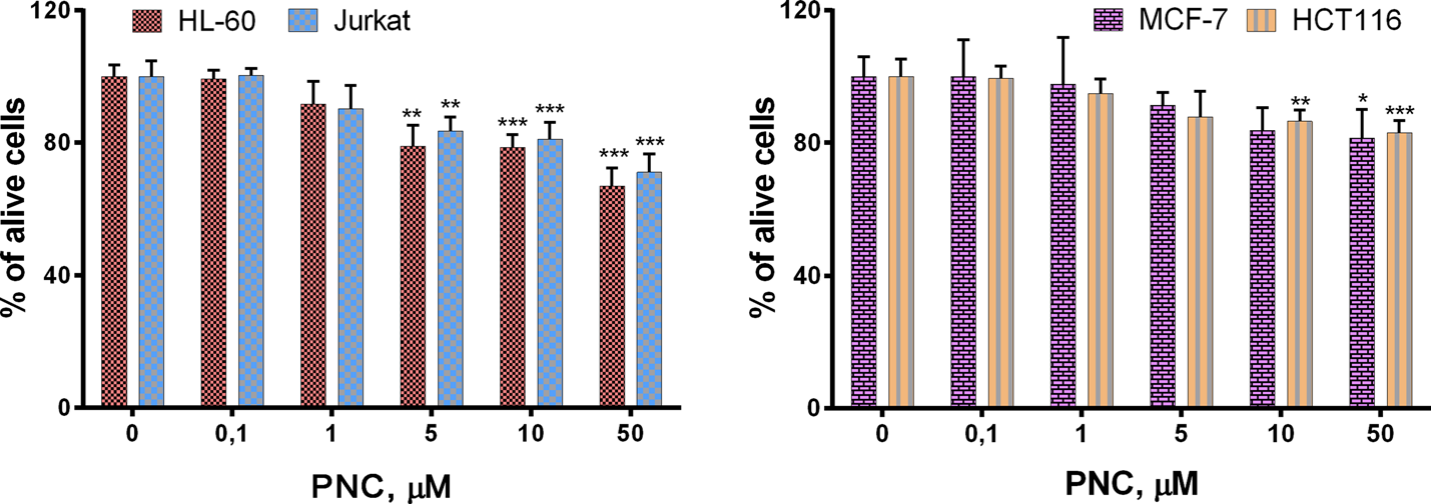
LC_50_ determined by Trypan blue exclusion test after treatment for 72 h of these cell lines with the PNC: human leukemia (HL-60, Jurkat), colon (HCT116), and breast (MCF-7) tumor. *P ≤ 0.05; **P ≤ 0.01 (difference compared with the non-treated control cells)

The results of the Trypan blue exclusion assay of the cytotoxic action of PNC were similar to the results of the MTT assay. We determined 66.9%, 71.1%, 81.4% and 83.0% of viable HL-60, Jurkat, MCF-7 and HCT116 cells, respectively, after incubation with PNC at the 50 µM dose for 72 h (Fig. 6).

### Physiological effects of the PNC

Administration of PNC at the highest doses (1 ml for mice and 10 ml for rats) induced a short suppression of the animals’ physical activity due to the high volume of the injected drug. However, on the day after injection, there were no visible changes in the behavior or physiological status of the animals. The same results were obtained by re-injecting the PNC in rats (10.0 ml dose that corresponded to 9.9 mg of the PNC) and mice (1.0 ml dose that corresponded to 9.9 mg of the PNC).

**Fig. 7:**
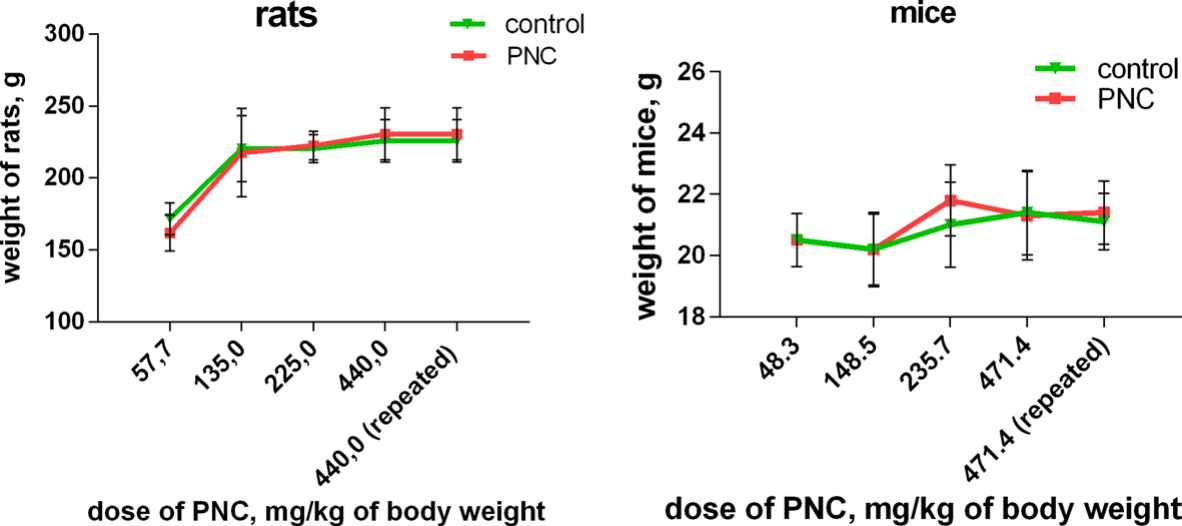
The body weight of rats and mice treated with the PNC used at different doses (abscissa)

The body weight of animals treated with the PNC at the indicated doses did not differ substantially from the body weight measured prior to the administration of the PNC or at the end of the experiment (Fig. 7). The administered doses of the PNC did not have a pronounced toxic effect on the animals, and no animal died during the study (Table 2); all animals survived during IP administration of PNC.

**Table 2:** The dosing of the polymeric nanocarrier (PNC) in the animals

Number of animals	Dose of PNC, mg/kg of body weight (ml of injected solution)	Dose of PNC, mg/kg of body weight (ml of injected solution)
	Rats	Mice
6	57.7 (1.0)	48.3 (0.1)
6	135 (3.0)	148.5 (0.3)
6	225 (5.0)	235.7 (0.5)
6	440 (10.0)	471.4 (1.0)
6 (repeat injection)	440 (10.0)	471.4 (1.0)

### Biochemical indicators of the PNC toxicity in vivo

The rats receiving the PNC demonstrated a significant 55.4% elevation in the activity of ALP on day 20 compared with control rats. The activity of other enzymes was close to the normal values with only minor deviations compared to the control group: GGT activity was decreased by 10%, ALT by 18%, LDH by 33%, and creatine phosphokinase by 16%, while α-amylase activity was 17% higher. Concentrations of blood serum metabolites remained mostly unchanged during PNC injections for 20 days (Table 3).

**Table 3:** Concentration of metabolites and activity of enzymes in blood serum of rats treated with the polymeric nanocarrier (PNC) for 20 days

	Control group	PNC treated group, 20 days
Aspartate transaminase activity, µkat/l	0.648 ± 0.045	0.538 ± 0.042*
Alanine transaminase activity, µkat/l	0.518 ± 0.034	0.425 ± 0.041*
De Ritis coefficient	1.25 ± 0.05	1.27 ± 0.09
γ-Glutamyltransferase activity, µkat/l	0.285 ± 0.034	0.255 ± 0.21
Creatine phosphokinase activity, µkat/l	2.31 ± 0.02	1.94 ± 0.05*
Alkaline phosphatase activity, µkat/l	2.58 ± 0.34	4.01 ± 0.28*
Lactate dehydrogenase activity, µkat/l	5.48 ± 0.12	3.69 ± 0.07*
α-Amylase activity, µkat/l	2.01 ± 0.09	2.36 ± 0.13
Total protein, g/l	76.5 ± 3.5	67.5 ± 3.2
Glucose, mmol/l	5.18 ± 0.89	6.09 ± 0.78
Urea, mmol/l	4.3 ± 0.2	5.1 ± 0.3
Creatinine, μmol/l	82.5 ± 4.1	70.7 ± 2.9*
Calcium cation, mmol/l	2.50 ± 0.34	3.22 ± 0.51
Iron cation, μmol/l	42.4 ± 2.1	42.5 ± 3.2
Sodium cation, mmol/l	127.5 ± 14.5	141.0 ± 9.8
Chloride anion, mmol/l	111.1 ± 4.9	119.8 ± 12.8

* P ≤ 0.05

The rats treated for 20 days with free PNC (i.e., lacking the conjugated anticancer compound) did not demonstrate significant changes in the concentration of total protein, urea, creatinine, or glucose in blood serum. The concentrations of calcium ions and iron also remained without substantial changes. Free PNC had no effect on the concentration of sodium cations and chlorine anions in blood serum. No nephrotoxic effect was recorded, highlighting the biosafety of this polymeric carrier used for delivery of anticancer drugs (Table 3).

Chemical components of the PNC were examined in urine and stool of rats treated with the PNC. The obtained results suggested that the PNC was metabolized, probably in liver tissue, and its clearance was under the control of the kidney. When estimating clearance of the PNC in daily urine and feces, it was found that rat feces contained 8–10% of the PNC in the native form, while urine contained 20% in the native form, with 15–20% as VEP and 13–25% as PEG. At the same time, liver tissue contained only traces of these products. Since all components of the PNC, namely, VEP, PEG, and PNC were present in the urine of the animals, the clearance of the degraded PNC likely occurred via filtration in the kidneys.

## Discussion

The polymeric nanoparticles are perhaps the simplest form of soft materials used for biomedical applications due to their facile synthesis and wide applicability for biotechnology and for diagnostics and treatment in medicine. The best-known class of the polymeric nanomedicines is based on utilization of single polymeric chains either directly as the therapeutic agent, or as a modifying agent for a drug. The complexation of many drugs can significantly improve the biomedical and physicochemical characteristics of these drugs. A review article published in 2016 identified 51 FDA-approved nanomedicines and 77 products in clinical trials (Bobo et al. 2016). Since 1990, 12 PEGylated drugs have been approved in the USA and/or Europe (Turecek et al. 2016). There are already several FDA-approved polymeric nanomedicines including glatiramer acetate for multiple sclerosis and leuprolide acetate for advanced prostate cancer (Johnson et al. 1998; reviewed in Bobo et al. 2016).

In the process of PEGylation, PEG polymer chains can be attached to molecules, such as a drug, or to other polymers to make them less hydrophobic. PEGylation can significantly increase the biological half-life of a substance in plasma (Benbrook 2015). The PNC used in our study also contains PEG side chains. Although ethylene glycol can produce negative effects in the body such as metabolic acidosis, nephrotoxicity, and disturbed electrolyte balance, the polymeric form of ethylene glycol (PEG) demonstrates low toxicity, and a dose of 10 mg/kg of animal body weight is acceptable for treatment (Bunker 2012; Feng et al. 2015). Some of these groups that are available in the structure of the polymeric side chains might be subjected to metabolic transformation in liver (enzymatic cleavage with participation of alcohol dehydrogenase), while the remainder (20–50%) might be excreted unchanged in the urine (Bunker 2012; Feng et al. 2015).

PNC used might produce peroxide groups (–O–O–) in the treated animals (Riabtseva et al. 2012). Previously, we have shown high thermal stability of the ditertiary peroxide groups with the activation energy of the peroxide group decomposition equal to 110 kJ/mole (Riabtseva et al. 2012). It should be noted that the content of the peroxide groups in the structure of the used PNC did not exceed 1% (Zaichenko et al. 1997, see Table 1). Besides, if these groups were not stable, we would expect changes in the enzymatic activities in the experimental animals. However, we did not observe such changes in blood serum of the treated animals (Kobylinska et al. 2015, 2016).

Liver contained only traces of these products that suggests very low or no accumulation of the PNC products in this organ. The feces of treated animals contained low concentration (8–10%) of PNC. The urine had twice higher PNC in native form and contained the fragments of the injected PNC. Since all components of the PNC (VEP, PEG, and PNC) were detected in the urine of treated animals, one can suggest that the clearance of the degraded PNC took place via kidney filtration.

In previous studies, we demonstrated an enhanced antineoplastic activity of the anticancer substances in mammalian tumor cells, when these substances were delivered by the developed PNC (Kobylinska et al. 2015, 2016). In this study, even at the highest concentrations these PNCs (the dose equivalent to the amount of polymer used in a complex with the anticancer drug) had a very weak toxic effect on cell viability. The data of this investigation together with the results of studying general toxicity of the free PNC (Kobylinska et al. 2015, 2016) suggest the biocompatibility of the PNC and confirm its potential for use as a drug delivery system (Heffeter et al. 2013; Senkiv et al. 2014).

## Conclusion

In this study, we demonstrated that the synthesized comb-like PEG-containing PNC possesses weak toxic effects toward human leukemia cells (HL-60 and Jurkat lines), as well as toward hepatocellular (HepG2), colon (HCT116), and breast (MCF-7) tumor cell lines. In addition, the PNC has no toxic effect in laboratory white mice (used at a dose of 470 mg/kg body mass, volume 1 ml) and laboratory Wistar rats (used a dose of 440 mg/kg body mass, volume 10 ml). Taking into account the obtained results, the PNC we developed and studied can be qualified as a compound of grade 4 toxicity (low toxicity substance). The reduced toxicity of this PNC in combination with its improved bioavailability and previously detected capability to enhance cytotoxicity toward tumor cells in vitro and tumor treatment effects in mice suggest great potential for its safe use as a drug delivery platform for treatment of various diseases, especially cancer.

## Abbreviations

ALP: alkaline phosphatase
ALT: alanine transaminase
AST: aspartate transaminase
DMSO: dimethyl sulfoxide
GGT: γ-glutamyltransferase
GMA: glycidyl methacrylate
LDH: lactate dehydrogenase
PEG: polyethylene glycol
PNC: polymeric nanocarrier
SEM: scanning electron microscopy
TEM: transmission electron microscopy
VEP: 5-(*tert*-butylperoxy)-5-methylhex-1-en-3-yne
